# Foot Weight-Bearing in Supported Standing: Influence of Verticalization Angles and Hip/Knee Flexion in Children and Adolescents with Cerebral Palsy (GMFCS IV-V)

**DOI:** 10.3390/children13030346

**Published:** 2026-02-27

**Authors:** Eva M. Steindl, René Althaus

**Affiliations:** 1 Independent Researcher, Schulstraße 5, 3300 Amstetten, Austria; 2 Independent Researcher, Auf der Seppe 19, 32689 Kalletal, Germany; r.althaus@gmx.net

**Keywords:** cerebral palsy, supported standing, plantar loading, foot weight-bearing, verticalization angle, hip and knee flexion, force plates, GMFCS, orthopaedic biomechanics, standing therapy

## Abstract

**Highlights:**

**What are the main findings?**

- Plantar loading increases progressively with higher verticalization angles, and clinically relevant loading (>70% body weight) is already achieved at approximately 60° verticalization in most hip/knee flexion conditions.
- Hip and knee flexion does not result in a linear reduction in plantar loading; substantial loading is maintained even at 45° flexion, with the highest loading observed at 90° verticalization and 30° hip/knee flexion.

**What is the implication of the main finding?**

- Effective and clinically meaningful plantar loading can be achieved in children and adolescents with severe cerebral palsy even in the presence of relevant hip and knee flexion, thereby enabling effective foot weight-bearing and supporting flexible and individualized standing positioning strategies.
- When prescribing and adjusting supported standing systems, the verticalization angle should be prioritized as the primary determinant of plantar loading, while joint flexion should not be considered a contraindication for effective foot weight-bearing.

**Abstract:**

**Background:** Supported standing is commonly prescribed for children and adolescents with cerebral palsy (CP) to support musculoskeletal health and participation. However, objective data on plantar loading under different positioning conditions are limited, particularly in individuals with severe motor impairment (GMFCS IV–V). This study quantified plantar loading as an operational measure of foot weight-bearing during supported standing across combinations of verticalization angle and hip/knee flexion. **Methods**: Twenty-six children and adolescents with CP (GMFCS IV–V; 6–17 years) were assessed using a standardized back-supported standing system. Plantar loading was measured with two calibrated force plates at six verticalization angles (0°, 30°, 45°, 60°, 75°, 90°) combined with four hip/knee flexion angles (0°, 15°, 30°, 45°). Loading was expressed as a percentage of body weight (% BW). Effects were analyzed using repeated-measures analysis of variance. **Results:** Plantar loading increased progressively with increasing verticalization angles across all hip/knee flexion conditions. Clinically relevant loading levels (>70% BW) were achieved at a verticalization angle of 60° in most flexion conditions. Maximum plantar loading was observed at 90° verticalization combined with 30° hip/knee flexion (96.4% BW). At 90° verticalization, plantar loading remained substantial even with 45° hip/knee flexion (81.4% BW). Increasing hip/knee flexion did not result in a linear reduction in plantar loading; a significant decrease was observed only at 45° flexion. **Conclusions**: Verticalization angle is the primary determinant of plantar loading during supported standing in children and adolescents with severe CP. Clinically meaningful plantar loading—and thus effective foot weight-bearing—can be achieved at moderate verticalization angles despite hip and knee flexion, supporting flexible positioning strategies.

## 1. Introduction

Cerebral palsy (CP) is a leading cause of long-term motor impairment in childhood and is associated with significant limitations in postural control, mobility and functional weight-bearing capacity [1].

Children and adolescents classified as GMFCS levels IV–V rely on supported standing systems to achieve an upright position. Assisted standing is associated with positive effects on bone mineral density, gastrointestinal function, joint range of motion, respiratory function, and participation in daily activities [2,3,4,5].

Despite these reported advantages, the biomechanical stress that actually occurs during supported standing, especially at the level of the feet, has not yet been sufficiently investigated. Adequate plantar loading is crucial for bone health, joint alignment and the provision of appropriate mechanical stimuli. Children and adolescents with severe CP have structural and functional factors, such as hip/knee flexion contractures, altered muscle tone and reduced extensor strength, which may influence effective foot weight-bearing during supported standing [6,7].

Previous studies have assessed overall foot loading [8,9,10]; however, a systematic quantification of plantar loading across defined combinations of verticalization angles and hip/knee flexion angles is not yet available. A detailed understanding of these biomechanical interactions is necessary to optimize supported standing programs according to individual anatomical and functional characteristics.

The aim of this study was to systematically quantify plantar loading as an operational measure of foot weight-bearing in children and adolescents with severe CP (GMFCS levels IV–V) across six defined verticalization angles and four combined hip/knee flexion positions using integrated force plates.

Primary Objective: The primary objective of this exploratory cross-sectional study employing a within-subject design was to quantify plantar loading under systematically varied, predefined combinations of verticalization angles and hip and knee flexion angles in children and adolescents with severe CP (GMFCS levels IV–V).

Secondary Objective: The secondary objective was to analyze potential interaction effects between verticalization angle and hip/knee flexion on plantar loading and to examine whether increasing hip/knee flexion is associated with a linear reduction in plantar loading.

## 2. Materials and Methods

### 2.1. Study Design

An experimental cross-sectional design was applied to analyze plantar loading under predefined combinations of verticalization angles and hip/knee flexion. The study was conducted and reported in accordance with the STROBE guidelines for cross-sectional studies.

### 2.2. Participants

Twenty-six children and adolescents with a confirmed diagnosis of CP (GMFCS IV–V; age range 6–17 years) participated in the study.

### 2.3. Inclusion and Exclusion Criteria

The inclusion and exclusion criteria are presented in Table 1.

### 2.4. Recruitment

Participants were recruited between January and February 2025 from specialized educational and therapy centres in Germany. Eligible participants were identified by treating therapists according to predefined eligibility criteria and invited through written information provided to legal guardians. As recruitment was conducted within specialized centres and not population-based, a potential risk of selection bias cannot be entirely excluded. All measurements were conducted on site in the respective institutions using the standardized standing system.

### 2.5. Equipment and Measurement System

**Figure 1:**
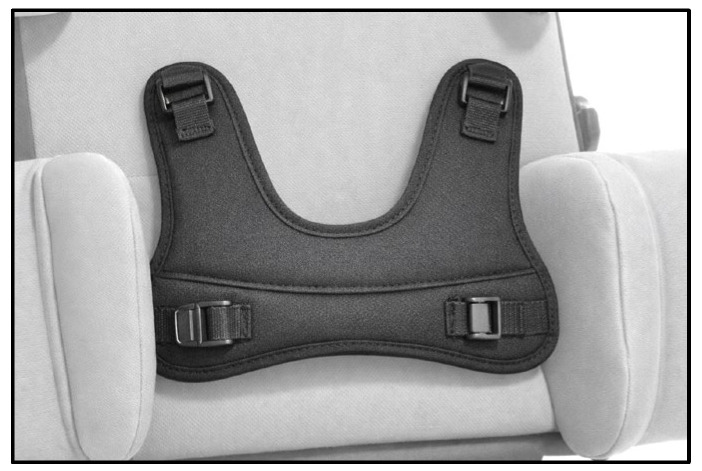
Shoulder support harness. Image provided by Schuchmann GmbH & Co. KG, Germany.

**Figure 2:**
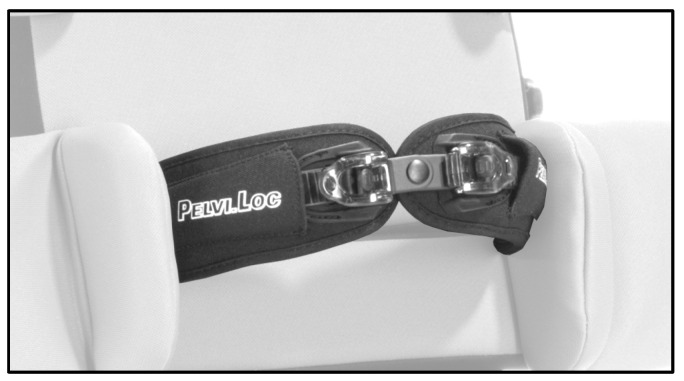
Pelvic strap. Image provided by Schuchmann GmbH & Co. KG, Germany.

Trained therapists transferred participants into a horizontally aligned back-supported reclining system. A patient lift was used when necessary, and the system was individually adjusted to each participant’s height and body proportions. Stabilization was achieved using thoracic and pelvic pads, supplemented by a pelvic belt and vest to ensure a safe and reproducible starting position (Figure 1 and Figure 2).

**Figure 3:**
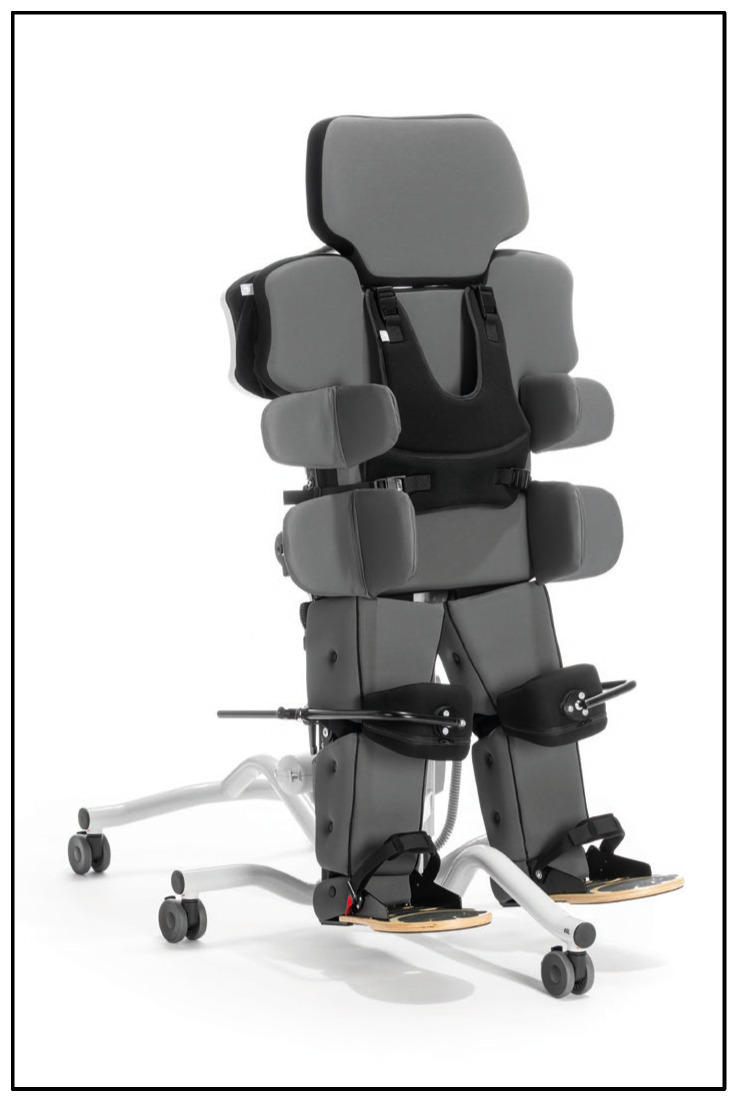
Till-supported standing system. Image provided by Schuchmann GmbH & Co. KG, Germany.

**Figure 4:**
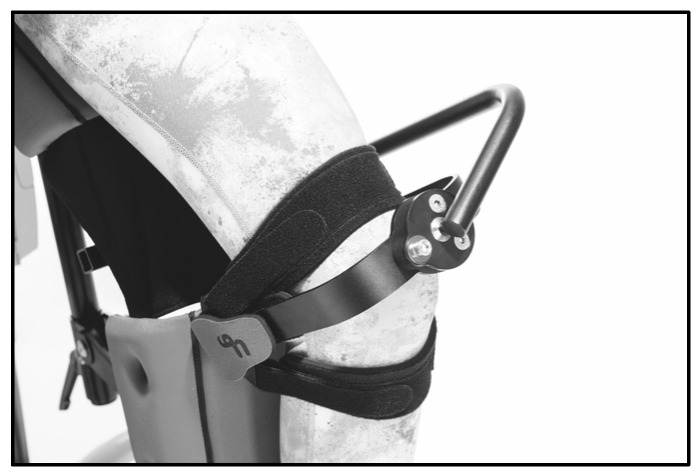
FKP knee pads. Image provided by Schuchmann GmbH & Co. KG, Germany.

The till-supported standing system (Schuchmann GmbH & Co. KG, Bissendorf, Germany) was used as the standing system (Figure 3). This system allows individually adjustable hip and knee flexion and provides uniform support for the torso and lower extremities via a continuous support surface. Individually adjusted knee pads (Figure 4) were used to reduce local pressure peaks and enhance comfort.

**Figure 5:**
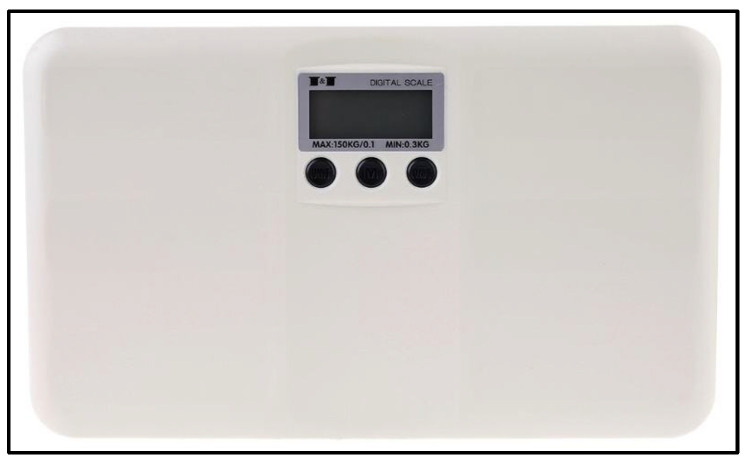
LX-02 Digital Travel Scale (generic OEM device manufactured in China). Source: Authors’ own photograph.

The feet were positioned flat on the footrests and aligned at a 90° angle relative to the lower legs, which were oriented parallel to the longitudinal axis of the torso. Plantar loading was measured using two digital scales (Figure 5).

Body weight was determined using a patient lift with integrated scales. All scales were calibrated using standardized weights prior to data collection to ensure measurement accuracy.

The verticalization angle was determined using a smartphone-based digital inclinometer application (Wasserwaage + Winkelmesser App, Version 1.3.8; WHATSTICKER APPS SRL, Milano, Italy) mounted on the standing system. Hip and knee joint angles were measured in a standardized manner using a goniometer aligned with the respective anatomical joint axes.

**Figure 6:**
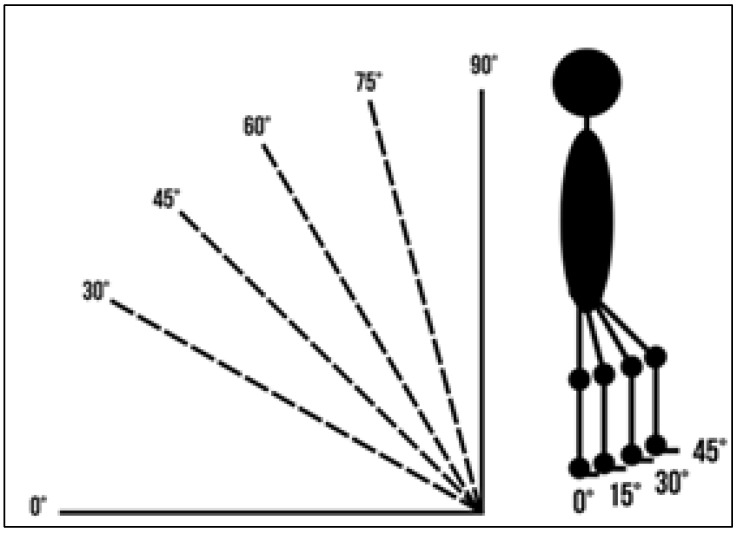
Illustration of the verticalization angles and hip/knee flexion used during the examination. Source: Authors’ own photograph.

Positions tested (Figure 6).

- Verticalization: 0°, 30°, 45°, 60°, 75°, 90°.
- Hip/knee flexion: 0°, 15°, 30°, 45°.

### 2.6. Procedure

**Figure 7:**
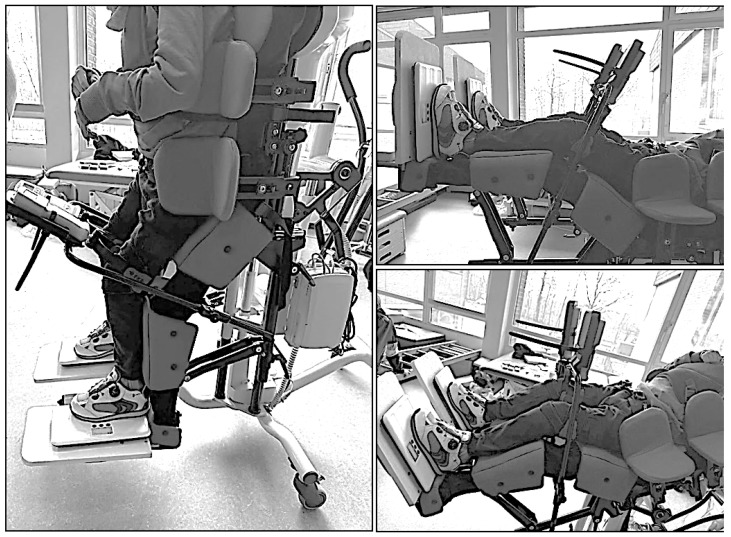
Positioning examples for HK 30° and V 90°, 30°, 0°. Source: Authors’ own photograph.

Participants were transferred into the back-supported standing system according to a standardized protocol performed by two trained pediatric therapists. All transfers were conducted using the Rebotec patient lift Arnold 150 (Rebotec Rehabilitationsmittel GmbH, Quakenbrück, Germany) with an integrated scale system to ensure a safe, controlled, and reproducible transfer from the wheelchair to the standing device. The transfer process and positioning within the back-supported standing system are illustrated in Figure 7.

The integrated scale of the Rebotec Arnold 150 was used to determine each participant’s body weight prior to positioning in the standing system. To ensure measurement accuracy and consistency between systems, the lift scale was calibrated before data collection using a standardized reference weight. The two digital foot scales used for plantar load assessment were calibrated using the same reference weight. This procedure ensured synchronization and comparability between the body weight measurement obtained via the lift and the vertical load values recorded at the feet.

Following transfer into the device, pelvic alignment was adjusted to achieve a neutral and symmetrical position. The pelvis was stabilized using a pelvic belt. Thoracic pads were individually adjusted to ensure upright trunk alignment, and a vest was applied when additional trunk stabilization was required. Care was taken to avoid pelvic rotation, lateral tilt, or excessive lumbar extension.

The lower extremities were positioned symmetrically, with the knees aligned in the sagittal plane. Individually adjusted knee pads were used to prevent excessive anterior translation of the tibia and to minimize localized pressure peaks. Particular attention was paid to maintaining axis-aligned positioning of the hip–knee–ankle chain.

All children used their existing, individually fitted orthoses or custom orthopedic footwear to ensure a standardized and biomechanically neutral foot and lower leg position. The feet were positioned flat on the footplates and aligned at approximately 90° relative to the lower legs. A neutral foot alignment without relevant pronation or supination was targeted. Footplate height and position were individually adjusted according to lower leg length and available joint range of motion.

The system was then gradually verticalized to the predefined angle. The predefined experimental positions, including hip/knee flexion and verticalization angles, are detailed in Table 2. During verticalization, therapists continuously monitored joint alignment, muscle tone, involuntary movements, and participant comfort to avoid abrupt loading, instability, or excessive spastic responses.

Before each measurement, an adaptation period of approximately 30 s was allowed to ensure postural stabilization and tone adaptation. Measurements were performed only after a stable and reproducible position had been confirmed by both therapists.

Each measurement condition was recorded once after confirmation of stability. In cases of involuntary movements, increased muscle tone, or visible instability, the measurement was repeated. The order of test conditions was identical for all participants due to safety and clinical feasibility considerations; this is acknowledged as a potential limitation.

Plantar loading was expressed as a percentage of body weight (%BW), calculated by normalizing the vertical load measured by the synchronized foot scales to the calibrated body weight obtained via the patient lift.

No adverse events or clinically relevant discomfort were documented during the transfer or measurement procedures.

### 2.7. Data Processing

The measurement data were exported and further processed to calculate mean values for each experimental condition. The percentage of body weight load (% BW) was calculated by normalizing the measured vertical load to the individual body weight of each participant. Data sets affected by movement artifacts, insufficient postural stability, or incomplete data recording were excluded from the analysis. Data collection took place between January and February 2025.

### 2.8. Statistical Analysis

To investigate the effect of verticalization angle (six levels) on foot load at each hip/knee flexion angle (four levels), separate repeated-measures analyses of variance (ANOVA) were performed. Greenhouse–Geisser or Huynh–Feldt corrections were applied when the assumption of sphericity was violated. The significance level was set at *p* < 0.05 (two-sided). Effect sizes were reported as partial eta squared (ηp^2^). All statistical analyses were performed using JASP (version 0.18.0) and DataTab (DATAtab e.U., Graz, Austria; available at https://datatab.net; accessed on 3 March 2025).

Sensitivity power analysis. A sensitivity power analysis was conducted using G*Power (version 3.1; F-tests: repeated-measures ANOVA, within factors) for the primary within-subject factor verticalization (six levels). The most conservative scenario was assumed (smallest available sample size n = 21; Greenhouse–Geisser nonsphericity correction ε = 0.32; correlation among repeated measures r = 0.50; α = 0.05; desired power = 0.80). Under these assumptions, the minimum detectable effect size was f = 0.348, corresponding to partial ηp^2^ = 0.108.

## 3. Results

Plantar loading increased progressively with increasing verticalization angle across all hip/knee flexion conditions. Separate one-way repeated-measures ANOVAs were conducted for each hip/knee flexion condition to examine the effect of verticalization (six levels) on plantar loading. A significant main effect of verticalization was observed in all flexion conditions (all *p* < 0.001), with large effect sizes (ηp^2^ = 0.86–0.91).

Descriptive statistics (mean ± SD and 95% confidence intervals) are presented in Table 3.

At 0° hip/knee flexion (n = 21), plantar loading increased from 26.6% BW (SD 16.14; 95% CI 19.25–33.95) at 0° verticalization to 87.79% BW (SD 20.56; 95% CI 78.43–97.15) at 90° verticalization (F(5,100) = 149.58, *p* < 0.001, ηp^2^ = 0.88).

At 15° flexion (n = 23), loading increased from 28.75% BW (SD 13.81; 95% CI 22.79–34.71) to 90.24% BW (SD 17.99; 95% CI 82.47–98.01) (F(5,110) = 223.09, *p* < 0.001, ηp^2^ = 0.91).

At 30° flexion (n = 23), plantar loading increased from 36.49% BW (SD 18.14; 95% CI 28.65–44.33) to 96.40% BW (SD 19.48; 95% CI 87.98–104.82) (F(5,110) = 159.05, *p* < 0.001, ηp^2^ = 0.88).

At 45° flexion (n = 25), loading increased from 29.07% BW (SD 9.08; 95% CI 25.32–32.82) to 81.35% BW (SD 21.65; 95% CI 72.41–90.29) (F(5,120) = 144.10, *p* < 0.001, ηp^2^ = 0.86).

Across flexion conditions, increasing hip/knee flexion did not result in a consistent reduction in plantar loading. At 90° verticalization, plantar loading was 87.79% BW at 0° flexion, 90.24% BW at 15° flexion, peaked at 96.40% BW at 30° flexion, and decreased to 81.35% BW at 45° flexion. This pattern suggests a non-linear influence of joint flexion on plantar loading.

**Figure 8:**
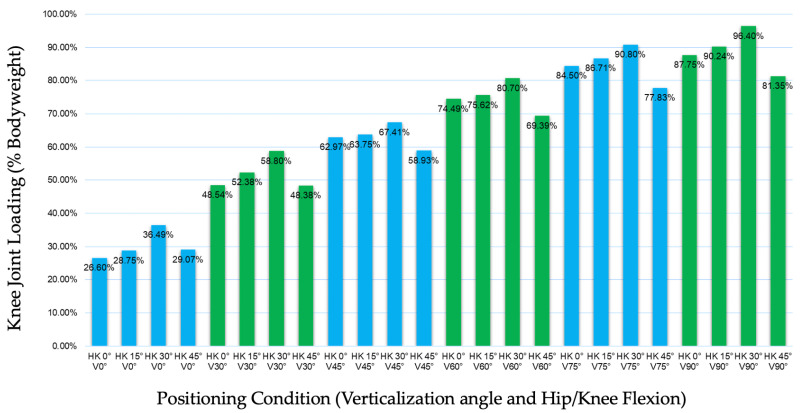
Percentage of plantar loading (%BW) across verticalization angle and hip/knee flexion conditions.

Clinically relevant plantar loading levels (>70% BW) were achieved at verticalization angles of 60° and above in most flexion conditions (Figure 8).

## 4. Discussion

To our knowledge, this is among the first studies to systematically investigate plantar loading in children and adolescents with severe CP (GMFCS IV–V) using predefined combinations of verticalization angles and hip/knee flexion during supported standing. The findings indicate that both the degree of verticalization and joint flexion are significantly associated with variations in the proportion of body weight transmitted through the feet.

While supported standing interventions are widely used in clinical practice, previous systematic reviews indicate that detailed biomechanical quantification of load distribution remains limited [11,12]. In the present study, the verticalization angle appeared to be the primary factor associated with increased plantar loading across all tested joint configurations. However, given the cross-sectional design, these findings must be interpreted as associative rather than causal.

Contrary to common assumptions in clinical practice, the presence of hip or knee flexion did not inherently prevent effective plantar loading. In several configurations, flexed joint positions maintained—and in certain combinations were associated with increased—plantar loading compared to full extension. The observation of increased loading at 30° hip/knee flexion at higher verticalization angles should be interpreted cautiously. From a biomechanical perspective, moderate knee flexion may alter lower limb alignment and the line of action of the ground reaction force, potentially influencing joint compression and load distribution. However, this explanation remains hypothetical and requires further investigation using biomechanical modeling approaches.

Previous studies examining weight-bearing in passive standers have reported that children with CP frequently demonstrate asymmetric right–left loading patterns [8,9,10].

Kecskemethy et al. further showed that different stander designs result in significantly different loading magnitudes [8]. Herman et al. demonstrated that actual weight-bearing in passive standers may be lower than expected and highly dependent on device configuration [9]. Importantly, these studies observed that load distribution remained relatively stable within a given standing configuration and changed primarily when inclination or device configuration was modified. This suggests that mechanical loading is largely determined by device settings and specific positioning within the system—particularly pelvic alignment, joint angles, axis alignment of the lower extremities, and foot placement—rather than by time-dependent adaptations during a single standing session [9].

Similarly, previous studies have demonstrated that inclination, hip abduction, device orientation, and muscle tone significantly influence weight-bearing in standing devices, with changes primarily associated with positional adjustments rather than spontaneous adaptations within an unchanged configuration. Changes in load distribution were primarily associated with positional adjustments rather than spontaneous adaptations within an unchanged configuration [10,11,12].

In the present study, mediolateral load distribution and right–left asymmetry were not analyzed separately, which represents a limitation. Although total plantar load was normalized to body weight, side-specific analyses could provide clinically relevant information, particularly in children with asymmetric tone, pelvic obliquity, or scoliosis. Future investigations should therefore incorporate continuous bilateral force measurements to better characterize asymmetrical loading patterns.

From a clinical perspective, the findings align with current recommendations for supported standing programs, which emphasize dosage but provide limited biomechanical guidance regarding optimal verticalization in the presence of contractures [3]. Systematic reviews report moderate yet clinically meaningful evidence for supported standing interventions, particularly with respect to bone mineral density and range of motion [11].

The present data suggest that clinically meaningful plantar loading (>70% BW) may be achievable at moderate verticalization angles (~60°), even in the presence of persistent hip/knee flexion. This is particularly relevant given the increased fracture risk and reduced bone mineral density frequently observed in children classified in GMFCS levels IV–V [11]. These children also demonstrate a high prevalence of hip displacement during growth [13,14].

Given the high prevalence of reduced bone mineral density, fracture risk, and progressive hip displacement in children classified in GMFCS levels IV–V, supported standing should be regarded as a core element of comprehensive postural management. In the evidence synthesis by Novak et al., weight-bearing and postural management interventions are classified as recommended within clinical practice frameworks [15]. Consistently, McLean et al. report that supported standing programs are most reliably associated with maintenance of bone mineral density and contracture prevention in non-ambulant children, despite heterogeneous evidence quality [11]. Moreover, Paleg et al. emphasize that upright positioning should be introduced developmentally “ON-Time,” potentially from 9–15 months of age. Together, these findings support early, individualized implementation of standing therapy rather than delayed initiation after secondary deformities have emerged [16].

Previous studies have further reported that weight-bearing in abduction and extension may positively influence hip stability in children with CP [17]. Randomized controlled trials have demonstrated that structured standing programs may improve or maintain bone mineral density in non-ambulant children with CP [2].

However, plantar loading should not be equated directly with osteogenic stimulation. Bone adaptation depends on multiple factors, including magnitude, frequency, rate of loading, and duration. The current findings therefore contribute to understanding mechanical exposure patterns but do not directly predict skeletal outcomes. Bone mechanotransduction is influenced not only by absolute load magnitude but also by strain rate and variability of mechanical stimuli [18]. Evidence from pediatric exercise research indicates that dynamic loading characteristics are critical determinants of bone adaptation [5].

A further implication concerns measurement technology. Integration of validatedforce-sensing systems within standing devices could enable precise, continuous monitoring of vertical and mediolateral loading in clinical practice. Such systems may support individualized adjustment of positioning and improve the detection of asymmetrical or potentially maladaptive loading patterns.

### 4.1. Clinical Implications

The results of this study have several clinically relevant implications:

- Children and adolescents with hip/knee flexion contractures were able to achieve clinically meaningful plantar loading within the tested configurations.
- Clinically relevant plantar loading levels (>70% of body weight) were observed at moderate verticalization angles (~60°) in this cohort [3].
- Individual adjustment of the standing system to the child’s anatomical alignment and available range of motion may help optimize plantar loading while supporting safe foot weight-bearing.
- When prescribing and adjusting standing devices, both the achievable verticalization angle and existing movement restrictions should be systematically considered to facilitate effective foot weight-bearing through optimized plantar loading.

### 4.2. Strengths and Limitations

Strengths of this study include the systematic investigation of 24 predefined combinations of verticalization angles and hip and knee flexion positions, the standardised positioning of participants, and the integration of force measurement within the standing system, allowing for consistent and reproducible assessment of plantar loading as an operational measure of foot weight-bearing.

Several limitations should be acknowledged. First, the measurements were limited to static standing positions, and dynamic transitions were not assessed. Second, the medio-lateral force distribution was not analysed. Finally, the study population was restricted to children and adolescents with GMFCS levels IV–V, which may limit the generalisability of the findings to less severely affected populations. In addition, separate repeated-measures ANOVAs were conducted for each flexion condition. Consequently, formal statistical inference regarding interaction effects between verticalization and flexion angles was not performed. Related observations should therefore be interpreted descriptively.

### 4.3. Future Research

Future research should focus on longitudinal studies investigating bone health and joint-related adaptations associated with supported standing and sustained foot weight-bearing. In addition, the influence of orthotic use, existing joint contractures, and different standing system designs on plantar loading patterns should be systematically examined. Further studies should also address dynamic transitions between different stages of verticalization and hip/knee flexion to better reflect clinically relevant weight-bearing conditions.

Given the exploratory design and the relatively small and clinically heterogeneous sample, the findings should be interpreted with caution and require confirmation in larger, controlled studies.

## 5. Conclusions

Higher verticalization angles are associated with a significant increase in plantar loading during supported standing. The presence of hip/knee flexion does not necessarily reduce plantar loading and may, depending on the configuration, maintain or even increase force transmission to the feet. Clinically relevant plantar loading levels can be achieved at moderate verticalization angles of approximately 60°.

These findings highlight the importance of precise and individually tailored adjustment of standing systems. Considering both verticalization angle and hip/knee flexion may contribute to more favorable load transfer through the feet and could support effective foot weight-bearing, particularly in children and adolescents with a restricted range of motion.

## Figures and Tables

**Table 1:** Inclusion and exclusion criteria.

Inclusion Criteria	Exclusion Criteria
Diagnosis of cerebral palsy (CP)	Other neurological disease
GMFCS Level IV or V	GMFCS Level I-III
Regular participation in supported standing therapy	Absence of regular standing therapy
Age 4–18 years	Age < 4 years or >18 years
	Lack of compliance or inability to follow study procedures
	Botulinum toxin injections to the lower extremities within the previous 6 months
	Orthopaedic surgery of the lower extremities within the previous 6 months
	Acute illness at the time of assessment
	Acute pain affecting standing tolerance or foot weight-bearing

**Table 2:** Positions and corresponding hip/knee flexion and verticalization angle.

Position	Hip Flexion Angle	Knee Flexion Angle	Verticalization Angles
1	0°	0°	0°, 30°, 45°, 60°, 75°, 90°
2	15°	15°	0°, 30°, 45°, 60°, 75°, 90°
3	30°	30°	0°, 30°, 45°, 60°, 75°, 90°
4	45°	45°	0°, 30°, 45°, 60°, 75°, 90°

**Table 3:** Plantar loading expressed as percentage of body weight (%BW) across verticalization angles at different hip/knee flexion angles. Values are presented as mean ± SD and 95% confidence intervals.

Hip/Knee Flexion	Verticalization	Mean (%BW)	SD	95% CI
0° (n = 21)	0°	26.60	16.14	19.25–33.95
	30°	48.54	12.74	42.74–54.34
	45°	62.97	11.86	57.57–68.37
	60°	74.49	14.23	68.01–80.97
	75°	84.50	17.67	76.46–92.54
	90°	87.79	20.56	78.43–97.15
15° (n = 23)	0°	28.75	13.81	22.79–34.71
	30°	52.38	13.33	46.62–58.14
	45°	63.75	13.06	58.11–69.39
	60°	75.62	14.66	69.29–81.95
	75°	86.71	18.56	78.69–94.73
	90°	90.24	17.99	82.47–98.01
30° (n = 23)	0°	36.49	18.14	28.65–44.33
	30°	58.80	16.49	51.68–65.92
	45°	67.41	18.10	59.59–75.23
	60°	80.70	17.86	72.98–88.42
	75°	90.80	17.27	83.34–98.26
	90°	96.40	19.48	87.98–104.82
45° (n = 25)	0°	29.07	9.08	25.32–32.82
	30°	48.38	11.55	43.61–53.15
	45°	58.93	13.25	53.46–64.40
	60°	69.39	17.20	62.29–76.49
	75°	77.83	19.19	69.91–85.75
	90°	81.35	21.65	72.41–90.29

## Data Availability

The data presented in this study are available from the corresponding author upon reasonable request. Data availability is subject to ethical restrictions due to the inclusion of sensitive pediatric patient data.
